# Inhibition of Porcine Epidemic Diarrhea Virus Replication and Viral 3C-Like Protease by Quercetin

**DOI:** 10.3390/ijms21218095

**Published:** 2020-10-30

**Authors:** Zhonghua Li, Hua Cao, Yufang Cheng, Xiaoqian Zhang, Wei Zeng, Yumei Sun, Shuhua Chen, Qigai He, Heyou Han

**Affiliations:** 1 State Key Laboratory of Agricultural Microbiology, College of Veterinary Medicine, Huazhong Agricultural University, Wuhan 430070, China; lzh1990@webmail.hzau.edu.cn (Z.L.); CaoHua@webmail.hzau.edu.cn (H.C.); cyf.hzau.edu.cn@webmail.hzau.edu.cn (Y.C.); 632071039@webmail.hzau.edu.cn (X.Z.); aiyouwei@webmail.hzau.edu.cn (W.Z.); sym@webmail.hzau.edu.cn (Y.S.); zhuling@webmail.hzau.edu.cn (S.C.); 2 State Key Laboratory of Agricultural Microbiology, College of Science, Huazhong Agricultural University, Wuhan 430070, China

**Keywords:** porcine epidemic diarrhea virus, quercetin, 3C-like protease, antiviral, drug target

## Abstract

For the last decade, porcine epidemic diarrhea virus (PEDV) variant strains have caused severe damage to the global pig industry. Until now, no effective antivirals have been developed for the therapeutic treatment of PEDV infection. In the present study, we found that quercetin significantly suppressed PEDV infection at noncytotoxic concentrations. A molecular docking study indicated that quercetin might bind the active site and binding pocket of PEDV 3C-like protease (3CL^pro^). Surface plasmon resonance (SPR) analysis revealed that quercetin exhibited a binding affinity to PEDV 3CL^pro^. Based on the results of the fluorescence resonance energy transfer (FRET) assay, quercetin was proven to exert an inhibitory effect on PEDV 3CL^pro^. Since coronavirus 3CL^pro^ is an important drug target and participates in the viral replication process, quercetin should be developed as a novel drug in the control of PEDV infection.

## 1. Introduction

Porcine epidemic diarrhea (PED) has been the most important viral diarrhea disease in pigs. PED is caused by porcine epidemic diarrhea virus (PEDV), which is a member of the genus *Alphacoronavirus* in the family *Coronaviridae* and is characterized by vomiting, watery diarrhea, dehydration, and a high mortality in piglets [1]. In the past 10 years, new outbreaks of PED have swept through many countries in Asia and America, leading to huge losses in the global pig industry [2,3,4,5,6]. Vaccination is considered as the best way to prevent PED. However, due to the genetic variation of PEDV, the traditional vaccine cannot provide efficient protection for pigs against variant PEDV. This can be illustrated by the outbreak of PED in China in 2010. In view of the current difficulty in terms of the prevention of PED, new strategies should be developed.

Quercetin, which is a flavonoid molecule, is commonly found in vegetables, fruits, and Chinese herbs [7]. Previous studies have reported that quercetin possesses antioxidative [8], anticancer [9], antibacterial [10], and antiviral [11] effects. The mechanism of antiviral effects of quercetin differs from virus to virus. For example, quercetin was found to have an excellent antiviral effect against porcine circovirus (PCV) and porcine reproductive and respiratory syndrome virus (PRRSV) through inhibiting the expression of heat shock protein 70 (Hsp70) [12,13]. In addition, it was recently determined that quercetin inhibited influenza entry by its interaction with hemagglutinin (HA) [14]. Moreover, the NS2 and NS3 protease activity of hepatitis C virus (HCV) was inhibited by quercetin, resulting in the suppression of the viral infection [15,16].

The genome of PEDV is a single stranded positive-sense RNA and includes at least seven open reading frames (ORFs) [17]. The five ORFs at the 3′ domain encode four structural proteins, namely spike (S), envelope (E), membrane (M), and nucleocapsid (N) proteins, and a nonstructural protein ORF3. The S, M, and E proteins form the viral envelope, and the N protein together with the viral genome form the nucleocapsid, making the virion an enveloped and pleomorphic structure with a range in diameter of 95 to 190 nm. The two ORFs at the 5′ domain called ORF1a and ORF1b comprise approximately two-thirds of the PEDV genome and encode polyproteins that are cleaved by viral protease into intermediate and mature nonstructural proteins [18,19]. Coronavirus non-structural protein 5 (nsp5), which is also called 3C-like protease (3CL^pro^), is involved in viral polyprotein cleavage and plays an important role in viral replication [20]. Therefore, 3CL^pro^ has always been an important target for developing drugs to be used in anti-coronavirus therapy [21,22].

In the present study, we found that quercetin could suppress PEDV infection by inhibiting PEDV 3CL^pro^ activity. Due to its excellent anti-PEDV effect, we hope to develop quercetin as a novel drug to deal with the puzzle caused by PED. 

## 2. Results

### 2.1. Toxicity of Quercetin on Cells

**Figure 1:**
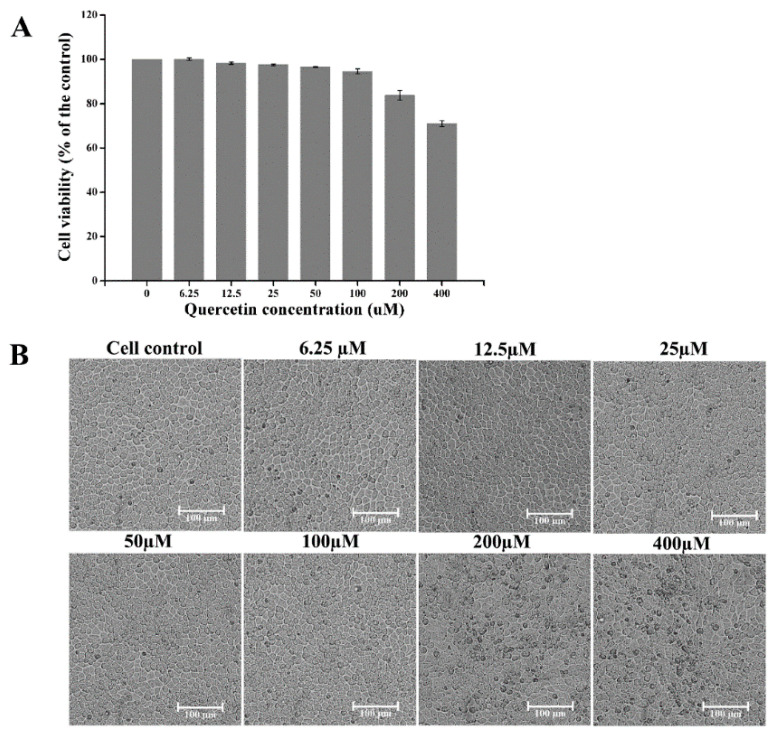
The cytotoxicity of quercetin on CCL-81 cells. (**A**) CCL-81 cell viability was measured by Cell Counting Kit-8 (CCK-8). All of the values were normalized to the cell control, which represents 100% cell viability. (**B**) CCL-81 cell morphology in the presence of quercetin at different concentrations. Data are shown as the means ± standard deviation (SD) of three independent experiments, with the error bars representing the standard deviations.

Since viral proliferation relies on the host cells, we needed to determine the concentrations of quercetin that are safe for the host cells in this anti-PEDV research. We evaluated the viability of CCL-81 cells treated by quercetin in the concentration range of 6.25 to 400 μM for 48 h. As shown in Figure 1, the cell viability was above 90%, and the cell morphology was not affected when treated with quercetin at a concentration of no more than 100 μM.

### 2.2. Quercetin Exhibits Antiviral Activity on PEDV

**Figure 2:**
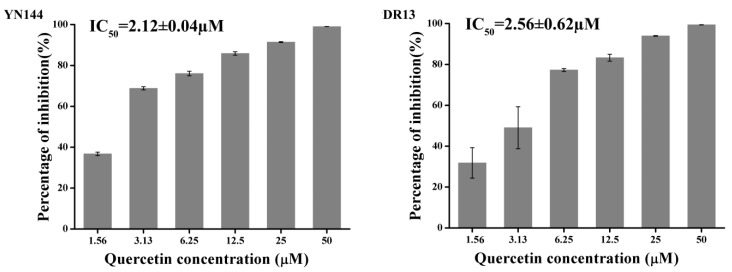
Antiviral activity of quercetin against the porcine epidemic diarrhea virus (PEDV) YN144 strain and DR13 strain in CCL-81 cells. CCL-81 cells grown on a 12-well plate were infected with the PEDV YN144 or DR13 strain at a multiplicity of infection (moi) of 0.001 in the presence of quercetin at different concentrations. Samples were collected at 24 hpi for the YN144 strain and 36 hpi for the DR13 strain and subjected to quantify the viral genome by quantitative real-time RT-PCR. The half maximal inhibitory concentration (IC_50_) was calculated by probit regression using SPSS Statistics 17.0 software to assess the inhibition ratios at different inhibitor concentrations.

Quantitative real-time RT-PCR was introduced to quantify the antiviral effects of quercetin on two PEDV strains. Based on the obtained results, quercetin exerted an obvious inhibitory effect on the production of the viral genome of PEDV YN144 and DR13 strain in a dose-dependent manner with a half maximal inhibitory concentration (IC_50_) of 2.12 ± 0.04 and 2.56 ± 0.62 µM, respectively (Figure 2, and Figures S1 and S2). The therapeutic index (TI) was greater than 100 (Supplementary Materials File S1), indicating the potential for developing quercetin as an anti-PEDV agent.

**Figure 3:**
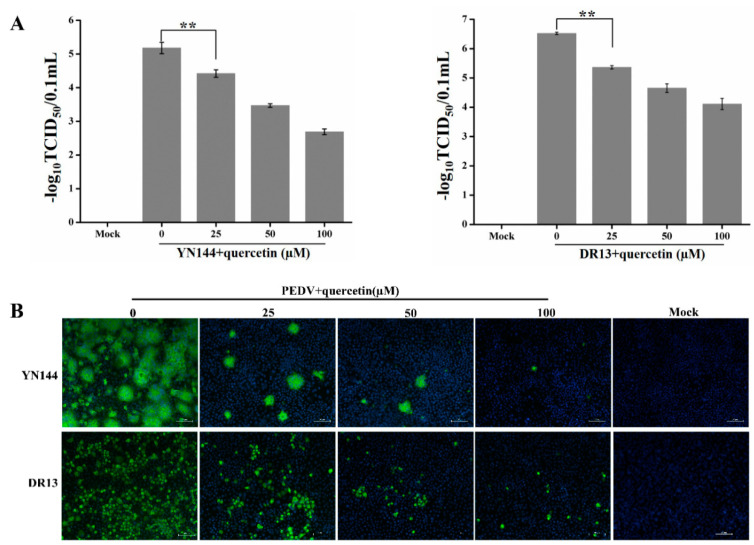
Measurement of the anti-PEDV effect of quercetin by TCID_50_ assay and indirect immunofluorescence assay (IFA). (**A**,**B**) CCL-81 cells were infected with the YN144 or DR13 strain in the presence of quercetin at the concentration of 0, 25, 50, and 100 μM. Samples were collected and used for the TCID_50_ assay and IFA. Data of the TCID_50_ are shown as the means ± SD of three independent experiments, with the error bars representing the standard deviations. ** represents a *p* value < 0.01.

To confirm the inhibitory effect of quercetin on YN144 infection, the TCID_50_ assay was conducted to determine quercetin’s inhibition of PEDV replication. As shown in Figure 3A, the titer of YN144 and DR13 significantly decreased with the increase of quercetin’s concentration. In addition, we also performed indirect immunofluorescence assay (IFA) to determine quercetin’s inhibition of the numbers of PEDV-infected cells. The IFA results demonstrated that quercetin could reduce the numbers of both YN144- and DR13-infected cells in a dose-dependent manner (Figure 3B). Moreover, less syncytium, the characteristic cytopathic effect (CPE) of YN144-infected cells, was formed in the quercetin-treated group, also proving the inhibition of quercetin in PEDV infection.

All of the data presented above adequately illustrate that quercetin could effectively inhibit PEDV infections in vitro and inspired us to further investigate the mechanism. 

### 2.3. Quercetin Could not Inhibit PEDV from Attaching to Cellular Membrane and Penetrating Cells

**Figure 4:**
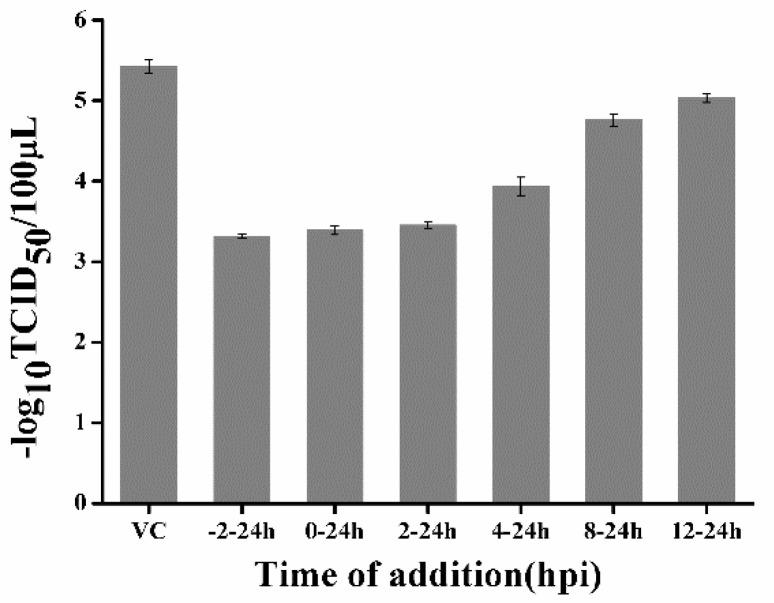
Time-of-addition effect of quercetin on PEDV replication in CCL-81 cells. Quercetin (50 μM) was added to infected culture at either before (−2–24 h), during (0–24 h), or after (2–24, 4–24, 8–24, and 12–24 h) PEDV infection. At 24 hpi, samples were collected and subjected to virus titration by TCID_50_ assay. VC: virus control; Data are shown as means ± SD of three independent experiments, with the error bars representing the standard deviations.

Inhibiting viral entry is one mechanism of quercetin anti-influenza virus [14]. In order to test quercetin’s effects on the entry of PEDV, a time-of-addition experiment was performed. For the current experiment, quercetin was added to the infected cultures at different time periods, and the reductions in virus yield, relative to those of untreated cultures, were determined at 24 hpi. As shown in Figure 4, when quercetin was present for the whole course of the replication cycle (−2 to 24 h), viral titer was significantly reduced, and the addition of quercetin during the 0–24 and 2–24 h stages exerted a similar effect, indicating that quercetin could not inhibit PEDV from entering cells. Moreover, adding quercetin during other periods following PEDV infection resulted in a gradual increase of viral yield, indicating the quercetin may exert its anti-PEDV effects through interference with the early events of viral replication.

### 2.4. Quercetin’s Anti-PEDV Effect is Independent of Its Hsp70 Inhibiting Activity

**Figure 5:**
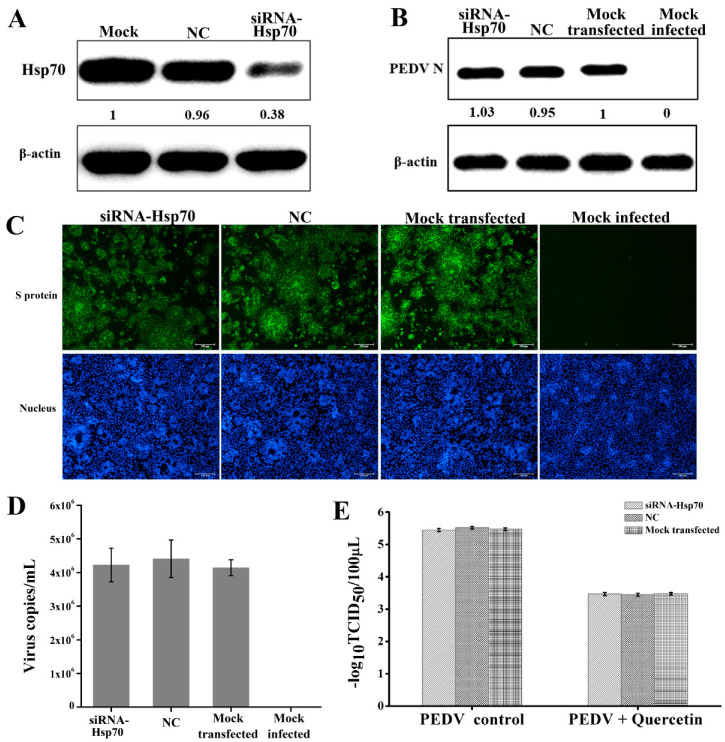
Analysis of the Hsp70 inhibiting activity of quercetin on PEDV replication. (**A**) CCL-81 cells transfected with siRNA against Hsp70 (siRNA-Hsp70), nonspecific control siRNA (NC), or without siRNA. Samples were collected at 48 h post transfection, and then expression of Hsp70 was analyzed by Western blot analysis. (**B**–**D**) CCL-81 cells transfected with siRNA against Hsp70, NC, or without siRNA for 48 h were infected with YN144 at moi of 0.001 or mock infected. Samples were collected at 24 hpi to analyze the expression of the PEDV N protein by Western blot analysis, display the numbers of PEDV-infected cells by IFA, and quantify the viral genome by quantitative real-time RT-PCR. (**E**) CCL-81 cells transfected with siRNA against Hsp70, NC, or without siRNA for 48 h and then infected with YN144 at moi of 0.001 in the presence (PEDV + quercetin) or absence (PEDV control) of 50 μM quercetin. Samples were collected at 24 hpi, and viral titer was measured with a TCID_50_ assay. Data of quantitative real-time RT-PCR and TCID_50_ assay are shown as the means ± SD of at least three independent experiments, with the error bars representing the standard deviations.

Admittedly, quercetin is a common inhibitor of Hsp70 [23,24]. Previous studies have proved that Hsp70 plays a positive role in the infection process of various viruses [12,13]. Consequently, we speculated that the anti-PEDV effect of quercetin may be due to its Hsp70 inhibiting activity. In order to test this, we silenced the Hsp70 by siRNA and then checked its influence on PEDV infection. As shown in Figure 5A, Hsp70 expression was successfully silenced by the siRNA against Hsp70. However, silencing Hsp70 did not result in a change in the expression of the PEDV N protein, the quantities of viral genome, and the numbers of PEDV-infected cells (Figure 5B–D), suggesting the absence of Hsp70 in PEDV infection in vitro. In addition, we also tested whether silencing Hsp70 could influence the effect of quercetin on PEDV replication. As shown in Figure 5E, silencing Hsp70 by siRNA before infection did not result in a change of quercetin’s effect on PEDV titer. Therefore, it could be concluded that the anti-PEDV effect of quercetin was independent of its Hsp70 inhibiting activity. 

### 2.5. Potential Sites of PEDV 3CL^pro^ Binding Quercetin

**Figure 6:**
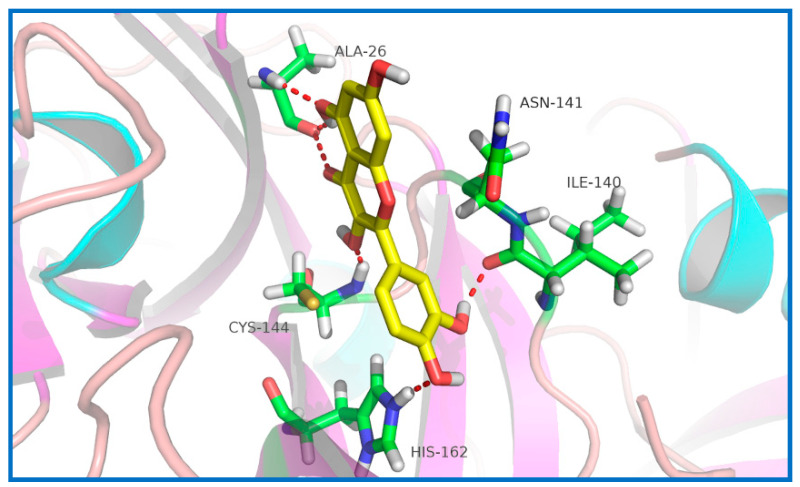
Docking of quercetin with PEDV 3C-like protease (3CL^pro^). Quercetin and amino acid residues contacting quercetin are represented as sticks. Quercetin and amino acid residues are colored according to different atom types. Moreover, along with the bond distance, hydrogen bond interactions are shown as red dashed lines between the respective donor and acceptor atoms.

Previous studies have demonstrated that several flavonoids could bind to some coronavirus’ 3CL^pro^ and inhibit its activity [21,25,26,27]. As a consequence, the interaction between quercetin and PEDV 3CL^pro^ was predicted by docking analysis. As shown in Figure 6, quercetin could interact with Cys144, Asn141, and His162. Therefore, quercetin is likely to block the recognition and combination of PEDV 3CL^pro^ and its substrates, which could thus inhibit PEDV replication.

### 2.6. Quercetin Showed Binding Affinity to PEDV 3CL^pro^

**Figure 7:**
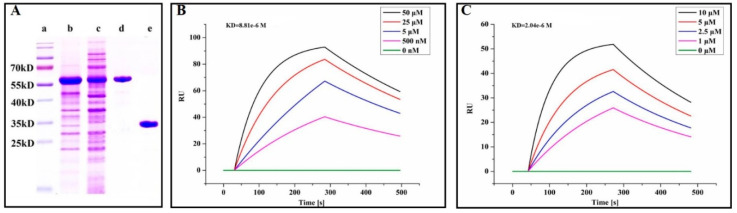
Analysis of the interaction between PEDV 3CL^pro^ and quercetin by surface plasmon resonance (SPR) assay. (**A**) Expression and purification of PEDV 3CL^pro^. Lane a, protein marker; lane b, inclusion body proteins; lane c, supernatant proteins; lane d, purified GST-tag PEDV 3CL^pro^; lane e, purified PEDV 3CL^pro^. (**B**) The binding curve of quercetin with 3CL^pro^ in the surface plasmon resonance (SPR) assay. (**C**) The binding curve of GC376 with 3CL^pro^ in the SPR assay.

In order to confirm the interaction between PEDV 3CL^pro^ and quercetin, the PEDV nsp5 gene was cloned into pGEX 6P-1 vectors for the expression of GST fusion proteins. Then, PEDV 3CL^pro^ was purified by cleaving the fusion proteins with PreScission protease (Figure 7A). The binding affinity between PEDV 3CL^pro^ and quercetin was measured by a surface plasmon resonance (SPR) assay. According to Figure 7B, quercetin increased the SPR signal in a significant and dose-dependent manner. Moreover, the dissociation constant (KD) for the interaction between quercetin and PEDV 3CL^pro^ was 8.18 μM. GC376, which is an inhibitor of coronavirus 3CL^pro^, was used as a positive control with a KD value of 2.04 μM in our assay (Figure 7C).

### 2.7. Quercetin Inhibits PEDV 3CL^pro^ Activity

**Figure 8:**
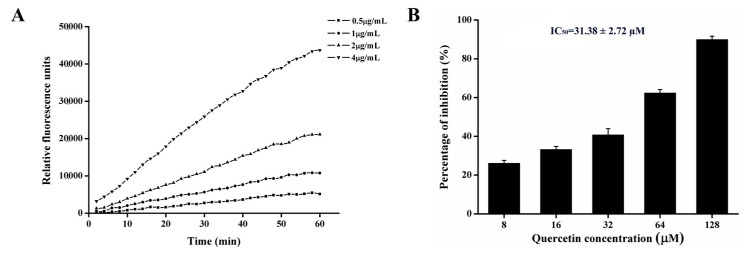
The inhibitory effect of quercetin on PEDV 3CL^pro^. (**A**) Analysis of the activity of the purified PEDV 3CL^pro^ in a fluorescence resonance energy transfer (FRET) assay. (**B**) Effect of quercetin on the activity of PEDV 3CL^pro^ in a FRET assay. IC_50_ was determined using SPSS 17.0 software. Data are shown as the means ± SD of three independent experiments, with the error bars representing the standard deviation.

In general, PEDV 3CL^pro^ recognizes a conserved cleavage site. Therefore, in order to test the cleavage activity of our purified protein, a fluorogenic peptide substrate was introduced in the fluorescence resonance energy transfer (FRET) assays. The fluorescence was increased with time after adding 3CL^pro^ into the substrate and with the increase of 3CL^pro^ (Figure 8A), suggesting that our purified protein could effectively cleave the fluorogenic peptide substrate [20]. As 3CL^pro^ plays an important role in PEDV replication, the inhibition of it must result in the reduction of PEDV infection. For the purpose of testing whether quercetin could inhibit the activity of PEDV 3CL^pro^, we introduced an inhibition activity assay by adding quercetin and PEDV 3CL^pro^ into the FRET system. As shown in Figure 8B, quercetin showed concentration-dependent inhibition, and the IC_50_ was 31.3 µM.

## 3. Discussion

The primary harm of PED is the high morbidity and mortality in piglets. Although a vaccine is an effective way to prevent PEDV infection, it has little effect after infection. Until now, no available drugs for PEDV have been developed and used in the clinic. As a result, there is an urgent need to develop novel anti PEDV agents to eliminate the threat of PED.

In the initial study, we found that quercetin displayed excellent antiviral activity against PEDV, which impelled us to investigate the mechanism of its anti-PEDV activity. It has been reported that quercetin has an antiviral effect in the infection process of many kinds of viruses. According to previous research, the mechanism of its antiviral effect could be summarized in the following three ways: (1) inhibiting the virus entering host cells [14]; (2) suppressing the expression of Hsp70 [12,13]; and (3) inhibiting the activity of viral protease [15,16,28,29,30]. We found that quercetin could not inhibit PEDV from attaching to and penetrating host cells and that Hsp70 was not involved in PEDV infection. Consequently, inhibiting the activity of PEDV protease seemed to be a possible approach for anti-PEDV quercetin uses.

Generally, 3CL^pro^ is a non-structural protein of PEDV that participates in the proteolytic procession during virus maturation [20,31]. The 3CL^pro^ of coronavirus has been reported as an attractive drug target in previous studies [32,33]. Screening inhibitors of coronavirus 3CL^pro^ is an advisable way of developing new drugs for viral therapy. Many flavonoids have been proved to be able to bind coronavirus 3CL^pro^ and inhibit its activity [21,22,26]. Chen et al. have reported that quercetin-3-b-galactoside, which is a derivative of quercetin, could also bind SARS-Cov 3CL^pro^ [34]. Speculating that quercetin could interact with PEDV 3CL^pro^, we found that quercetin could interact with Cys144, Asn141, and His162 of PEDV 3CL^pro^ based on docking analysis. According to related studies, the active sites of PEDV 3CL^pro^ are composed of His41 and Cys144, and the S1 specificity pocket consists of Phe139, Ile140, Asn141, Gly142, Ala143, Cys144, His162, Gln163, and Glu165 [20]. Therefore, there is a possibility that quercetin could inhibit the activity of PEDV 3CL^pro^ by binding to the active sites and S1 specificity pocket of PEDV 3CL^pro^. We used SPR to assess whether there was a binding affinity between quercetin and PEDV 3CL^pro^. The SPR result indicated that quercetin showed a medium binding affinity to PEDV 3CL^pro^. Although quercetin was proven to be able to bind PEDV 3CL^pro^, we still needed to test whether it could inhibit PEDV 3CL^pro^ activity. FRET was chosen to verify the inhibiting effect of quercetin on PEDV 3CL^pro^. According to the result of FRET, quercetin could effectively inhibit PEDV 3CL^pro^ activity. Although our study has demonstrated that quercetin could bind to PEDV 3CL^pro^ and inhibit its activity, the actual binding sites were not determined, as the crystal structure of the complex of PEDV 3CL^pro^ and quercetin has not been figured out by our laboratory to date. Related research is still in progress, since it is of importance for revealing the details of quercetin interaction with PEDV 3CL^pro^ and the development of new anti-PEDV agents according to the structure of quercetin.

PEDV variant strains have been prevalent globally, and severely affect and hinder the development of the pig industry. In the present study, we identified quercetin as a novel antiviral agent, which can supposedly be applied as a safe, effective, and cheap treatment of PEDV infection. Moreover, our result demonstrated that quercetin could effectively inhibit PEDV 3CL^pro^ activity, which was responsible for the suppression of PEDV. Our result will contribute to developing more chemical antiviral drugs targeting PEDV 3CL^pro^ in accordance with the structure of quercetin, which may be used in the therapy of PEDV infection.

## 4. Materials and Methods 

### 4.1. Virus, Cells, Antibodies, and Plasmid

The PEDV YN144 (GenBank accession No. KT021232) and DR13 (GenBank accession No. JQ023161) strains were used throughout the present study. CCL-81 cells were cultured in Dulbecco’s modified Eagle’s medium (DMEM) containing 8% fetal bovine serum (FBS) and incubated at 37 °C with 5% CO_2_. Virus titers were determined by calculating the 50% tissue culture infectious dose (TCID_50_) using the Reed–Muench method. Quercetin was purchased from Medchemexpress (Shanghai, China) with a purity of more than 99%. The rabbit polyclonal anti-Hsp70 antibody, mouse anti-β-actin mono-antibody (Mab) and HRP labeled antibodies were purchased from ABclonal (Wuhan, China). The Alexa 488-labeled anti-mouse antibody was purchased from Antgene (Wuhan, China). MAb against the PEDV N protein was purchased from Youlong Biotech (Shanghai, China). MAb against the PEDV S protein was developed by our laboratory.

### 4.2. Cytotoxicity Assay 

The cytotoxicity of quercetin on CCL-81 cells was measured by a Cell Counting Kit-8 (CCK-8; Glpbio, Montclair, CA, USA) following the manufacturer’s instructions. CCL-81 cells grown on a 96-well plate were treated with quercetin of different concentrations for 48 h. After being washed with PBS three times, 110 µL CCK8 solution (10 µL CCK8 regent in 100 µL DMEM) was left for 4 h at 37 °C with 5% CO_2_ in the dark. After being shaken on a table concentrator for 1 min, the optical density 450 (OD_450_) was measured by a microplate reader. Cell viability was calculated according to the following equation: cell viability = [As − Ab]/[Ac − Ab]. As: the OD_450_ value of the quercetin treated wells (wells with quercetin treated cells and CCK8 solution); Ab: the OD_450_ value of the mock wells (wells only with CCK8 solution); Ac: the OD_450_ value of the wells without quercetin (wells with cells and CCK8 solution).

### 4.3. Quantitative Real-Time RT-PCR 

RNA was extracted using Tripure Isolation Reagent (Roche, Indianapolis, IN, USA) following the manufacturer’s instructions. The cDNA was obtained by RT-PCR with the Primescript^TM^ RT Master mix (TakaRa, Shiga, Japan). The real-time RT-PCR assay for quantifying the PEDV genome used the following primer and probe sequences: PEDV forward primer: 5′-CGTACAGGTAAGTCAATTAC-3′; PEDV reverse primer: 5′-GATGAAGCATTGACTGAA-3′; and PEDV Taq-Man^®^ probe: FAM-TTCGTCACAGTCGCCAAGG-TAMRA. Primers employed for the relative RT-PCR were as follows: Hsp70-F: 5′-AGGAGTTCCATATCCAGAA-3′; Hsp70-R: 5′-CAGCTCGACATTCACCAC-3′; GAPDH-F: 5′-TGACAACAGCCTCAAGATCG-3′; and GAPDH-R: 5′-GTCTTCTGGGTGGCAGTGAT-3′.

### 4.4. TCID_50_ Assay

Virus stock solutions were serially diluted before they were inoculated on the confluent CCL-81 cell monolayers grown in the 96-well plates, followed by washing three times with PBS. Eight wells were inoculated with 100 μL at each dilution, and plates were incubated at 37 °C with 5% CO_2_ for 2 days. For the YN144 strain, wells with syncytium formation, the specific cytopathic effect caused by YN144, were classified as PEDV-positive. For the DR13 strain, after 2 days of incubation, plates were subjected to IFA staining, and wells with specific staining were considered to be PEDV-positive. PEDV titration was calculated by tissue culture infectious dose 50 (TCID_50_) following the Reed–Muench method established by L. J. Reed and H. Muench.

### 4.5. Indirect Immunofluorescence Assay

Cells grown on a 24-well plate were washed gently with PBS and then fixed with ice cold ethanol for 30 min at −20 °C. After being washed three times with PBS, cells were blocked in PBS containing 1% bovine serum albumin (BSA) for 30 min at room temperature. Cells were incubated with anti-PEDV S protein antibodies in PBS for 1 h at 37 °C, followed by three washes with PBS. Subsequently, cells were incubated with Alexa 488-labeled anti-mouse antibody (Antgene, Wuhan, China) for 45 min at 37 °C. After three washes, cells were treated with DAPI dihydrochloride (Beyotime Biotechnol, Shanghai, China) at room temperature for 5 min to stain the nuclei.

### 4.6. Western Blot 

Cells grown on the 6-well plate were washed gently with PBS and then lysed with lysis buffer (Beyotime Biotechnol, Shanghai, China) containing 1 mM phenylmethanesulfonyl fluoride (PMSF). After adding the loading buffer, cell lysates were boiled for 10 min. After being cooled on ice, samples were subjected to SDS-PAGE, followed by being blotted onto polyvinyl difluoride (PVDF) membranes. After the transmembrane process, the membranes were blocked with TBS buffer containing 0.05% tween-20 (TBST) and 5% skim milk for 2 h at room temperature. After blocking, the membranes were incubated with the primary antibodies for 2 h at room temperature, followed by being washed with TBST. Then the membrane was incubated with the corresponding HRP labeled secondary antibody for 1.5 h at room temperature. After being washed three times, the membranes were subjected to the Clarity™ Western ECL Blotting Substrate (Bio-Rad, Hercules, CA, USA) to visualize protein bands. 

### 4.7. Time-Of-Addition Assays

CCL-81 cells were infected with the PEDV YN144 strain at a moi of 0.001. Then, 50 μM quercetin was added into infected culture at different time periods of PEDV infection: −2–24, 0–24, 2–24, 4–24, 8–24, and 12–24 h. At 24 h post infection, culture supernatants and cell lysates were collected following freeze–thaw cycles and then subjected to virus titration by TCID_50_ assay.

### 4.8. RNA Interference

The siRNA designed to interfere with the expression of Hsp70 was synthesized by Gene Pharma (Shanghai, China). The sequence of the siRNA was 5′-CCAAGCAGACGCAGAUCUUTT-3′. CCL-81 cells were seeded into corresponding well plates and incubated at 37 °C for nearly 12 h before transfection. The siRNA was transfected into CCL-81 cells with lipofectamine 2000 (Invitrogen, Carlsbad, CA, USA) according to the manufacturer’s instructions.

### 4.9. Docking of PEDV 3CL^pro^ and Quercetin

The crystal structures of PEDV 3CL^pro^ (PDB: 4XFQ) and quercetin were downloaded from https://www.rcsb.org/. Homology models of PEDV 3CL^pro^ with quercetin were generated by Autodock 4 software. Grid maps of 52 × 56 × 50 grid points were produced, covering the active site and binding pocket of PEDV 3CL^pro^. The parameters for docking were a population size of 150, maximum of 5 million energy evaluations, maximum of 27,000 generations, mutation rate of 0.02, and crossover rate of 0.8. In total, 100 docked conformations were yielded, and the conformation with the lowest binding energy was selected for analyzing the interaction between quercetin and PEDV 3CL^pro^. Docking results were analyzed by PyMOL software to obtain the most likely binding pattern.

### 4.10. Protein Expression and Purification

The PEDV nsp5 gene was cloned into the pGEX 6p-1 vector for the expression of PEDV 3CL^pro^. The recombinant plasmid was transformed into *Escherichia. coli* BL21(DE3) and then cultured at 37 °C in LB medium until the OD_600_ reached 0.6–0.8. Afterwards, 1 mM isopropyl β-D-thiogalactoside (IPTG) was added to induce protein expression. The bacteria were harvested after incubation at 16 °C for 24 h, resuspended in PBS and disrupted. The supernatant was filtered and loaded onto a Glutathione Sepharose 4B column (GE Healthcare, Pittsburgh, PA, USA). Finally, the N terminal GST-tagged protein was eluted using elution buffer (10 mM Glutathione in 50 mM Tris-HCl, pH 8.0). The N-terminal GST tag proteins were digested with PreScission Protease (Beyotime, Shanghai, China) and loaded onto a Glutathione Sepharose 4B column so as to remove the GST tag and GST-tagged PreScission Protease. 

### 4.11. Surface Plasmon Resonance

To examine the binding of quercetin to PEDV 3CL^pro^, surface plasmon resonance (SPR) was performed on an OpenSPR system (Nicoya Lifesciences Inc., Kitchener, ON, Canada) according to the description by McGurn et al. [35]. PEDV 3CL^pro^ without a GST tag served as the ligand, which was immobilized on COOH-sensor chips. Subsequently, quercetin solution was applied into the sensor chip with PBS as the running buffer. The kinetic constants, including the association constant (ka), dissociation constant (kd), and affinity (KD, KD = kd/ka), were calculated with the software according to a 1:1 binding model.

### 4.12. Fluorescence Resonance Energy Transfer Assays for Enzymatic Characteristics

In order to test the inhibiting effect of quercetin on PEDV 3CLpro, a peptide substrate, Dabcyl-YNSTLQ↓AGLRKM-E-Edans, was designed according to the N-terminal cleavage site of PEDV 3CL^pro^. The two fluorophores formed a quenching pair and exhibited fluorescence resonance energy transfer (FRET) in the peptide. The enzyme reaction system (200 μL) for measuring PEDV 3CL^pro^ activity was composed of 10 μM FRET substrate, 20 mM Tris/HCl buffer (pH 7.5), and 3CL^pro^ of various concentrations. The fluorescence of reactions was monitored for 60 min at 340 nm excitation and 485 nm emission, using fluorescence plate reader reactions with the Spark 10M multimode reader platform (TECAN, Switzerland). The reaction system (200 μL) for the inhibition assay was composed of 1μg/mL 3CL^pro^, 10μM FRET substrate, quercetin of different concentrations, and 20 mM Tris/HCl buffer (pH 7.5). Quercetin at various concentrations was pre-incubated with 3CL^pro^ for 20 min at 37 °C, and FRET substrate was added to the mixture. In addition, a reaction system without quercetin was set as the control group. The fluorescence of reactions was monitored according to the method mentioned above. The inhibition ratio was calculated using the following equation: Percentage of inhibition (%) = 100 × [1 − fluorescence of the experimental group (60 − 0 min)/fluorescence of the control group (60 − 0 min)].

## Supplementary Materials

Supplementary materials can be found at https://www.mdpi.com/1422-0067/21/21/8095/s1.
