# Supplementary material for: Inhibition of Porcine Epidemic Diarrhea Virus Replication and Viral 3C-Like Protease by Quercetin
Source: Int J Mol Sci. 2020 Oct 30;21(21):8095. doi: 10.3390/ijms21218095 (PMC7662296; doi:10.3390/ijms21218095)
Supplement: Supplementary file 1 [file ijms-21-08095-s001.zip › Supplementary Materials/Supplementary Materials Figure S1.docx]

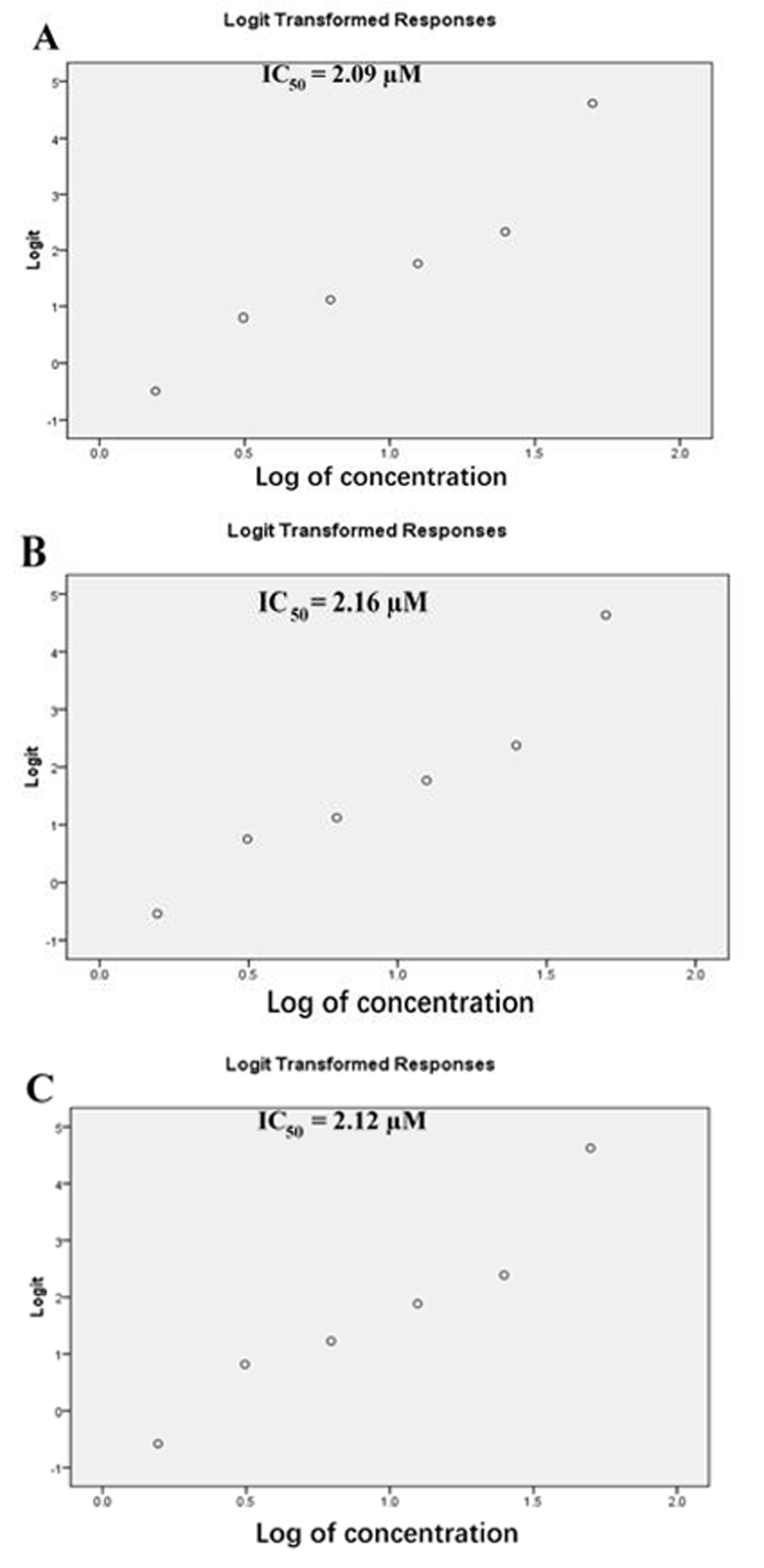


Figure S1. The curve-fitting used to determine the IC_50_ of quercetin inhibiting PEDV YN144 strain. The IC_50_ was calculated by probit regression of SPSS statistics 17.0 to assess the inhibition ratios at different inhibitor concentrations. The X axis represents the log of the inhibitor concentrations and the Y axis represents the log of the percentage of inhibition. A, B and C represent the result of three biological repeats
