# Supplementary material for: Inhibition of Porcine Epidemic Diarrhea Virus Replication and Viral 3C-Like Protease by Quercetin
Source: Int J Mol Sci. 2020 Oct 30;21(21):8095. doi: 10.3390/ijms21218095 (PMC7662296; doi:10.3390/ijms21218095)
Supplement: Supplementary file 1 [file ijms-21-08095-s001.zip › Supplementary Materials/Supplementary Materials Files S1.docx]

| CC_50_^a^ (μM) | IC_50_^b^ (μM) | | TI^c^ | |
| --- | --- | --- | --- | --- |
|  | YN144 | DR13 | YN144 | DR13 |
| > 400 | 2.12±0.04 | 2.56±0.62 | >185 | >125 |

Antiviral activity of quercetin against PEDV in CCL-81 cells.

a: Concentration required to reduce cell growth by 50%; b: Half maximal inhibitory concentration; c: Therapeutic index = CC_50_/IC_50_.
